# Assessment of multiplex *Onchocerca volvulus* peptide ELISA in non-endemic tropical regions

**DOI:** 10.1186/s13071-019-3824-x

**Published:** 2019-11-29

**Authors:** Ole Lagatie, Elodie Granjon, Maurice R. Odiere, Maan Zrein, Lieven J. Stuyver

**Affiliations:** 1 Janssen Global Public Health, Turnhoutseweg 30, 2340 Beerse, Belgium; 2 InfYnity Biomarkers, 78 rue du Bourbonnais, 69009 Lyon, France; 3 Kenya Medical Research Institute, Centre for Global Health Research, P. O. Box 1578, 40100 Kisumu, Kenya

**Keywords:** *Onchocerca volvulus*, Peptide serology, Diagnostics, Multiplex ELISA

## Abstract

**Background:**

Currently, serodiagnosis of infection with the helminth parasite *Onchocerca volvulus* is limited to the Ov-16 IgG4 test, a test that has limited sensitivity and suboptimal specificity. In previous studies, we identified several linear epitopes that have the potential to supplement the diagnostic toolbox for onchocerciasis.

**Methods:**

In this study three peptides, bearing in total six linear epitopes were transferred to a multiplex ELISA platform. This multiplex ELISA was used to assess the clinical utility of the peptide serology markers by analyzing sample sets from both *O. volvulus* endemic and non-endemic regions.

**Results:**

The multiplex platform was shown to be reproducible and data obtained on the multiplex platform were comparable to the singleplex ELISA data. The clinical utility assessment showed that in a population of school-aged children from western Kenya, a virtually *O. volvulus*-free area, significant cross-reactivity with an as-yet to be determined immunogen was detected.

**Conclusions:**

The observations made in this study invalidate the usefulness of the peptide serology markers for onchocerciasis detection. We discuss what could be the origin of this unexpected serological response, but also highlight the need for better characterized biobanks for biomarker discovery activities.
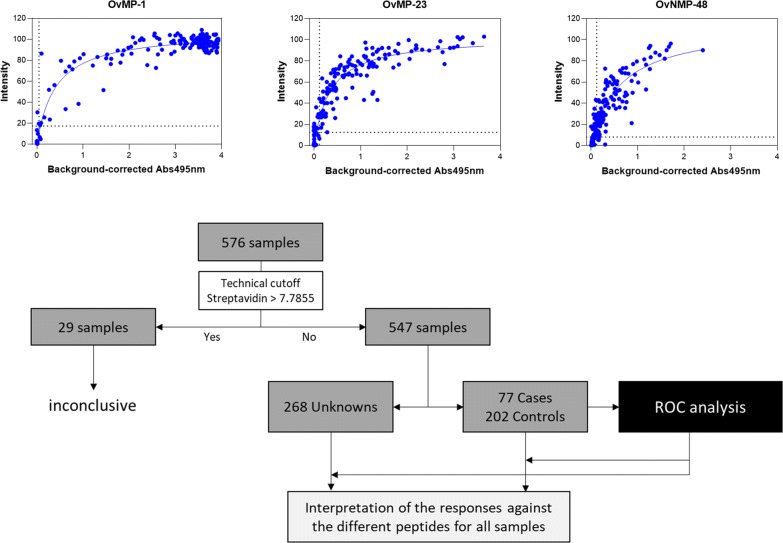

## Background

Helminth infections make up a major part of the diseases listed on the World Health Organization (WHO) list of Neglected Tropical Diseases (NTD) [1–3]. One of these helminths, *Onchocerca volvulus* causes onchocerciasis (also known as river blindness). At least 120 million people are at risk of infection, the majority of them living in Africa [4, 5]. Efforts to control the spread of the disease are centered around mass drug administration (MDA) of the microfilaricidal agent ivermectin (Mectizan, Merck). *Onchocerca* infection is traditionally diagnosed by microscopic detection of microfilariae (mf) in skin biopsy samples (skin snips) and by serological assays detecting IgG4 antibodies against the Ov16 antigen [6, 7]. WHO guidelines for stopping MDA and verifying elimination of onchocerciasis require demonstration of < 0.1% seroprevalence of Ov-16 IgG4 in children under the age of 10 years [8]. The specificity of the Ov-16 rapid diagnostic test (RDT) ranges from 97% to 98%, which makes it virtually impossible to achieve the 0.1% threshold, and hence causes failure in recognizing interruption of transmission and stopping MDA [9–11].

One way to overcome the specificity challenge of the Ov16 IgG4 test, would be to introduce a serological confirmation test to confirm or refute a positive test result in a first line screening assay. The combination of peptides OvMP-1, OvMP-23 and OvNMP48 in a peptide-based multiplex ELISA test could possibly provide such second line confirmation capabilities [12–14].

In this study we demonstrate that a multiplex ELISA using these *Onchocerca*-derived peptides in the InfYnity Biomarkers platform confirms the previously demonstrated sensitivity [15, 16]. The clinical utility of these peptide antigens was not proven, since significant cross-reactivity with an as-yet to be determined immunogen was detected in a population of school-aged children in a virtually *O. volvulus*-free area of western Kenya. This observation invalidates the usefulness of these peptides as antigens to build a confirmation test for the Ov16 IgG4 RDT.

## Methods

### Study samples

Plasma samples from *O. volvulus* infected individuals, healthy controls, *W. bancrofti-*infected individuals, individuals with *B.* *malayi* infection and individuals infected with soil-transmitted helminths (STH) and/or *Schistosoma mansoni* have been described before [12, 13, 17]. An overview, including Ov16 IgG4 information is provided in Table 1.

**Table 1:** Study populations included in this study

Group	*n*	Origin	Helminth infection status	Ov16 IgG4-positive (%)
Nodule-positive	84	Ghana, Ashanti	*O. volvulus-*infected	55 (65.5)
*B. malayi*	20	Indonesia, Central Sulawesi	*B. malayi-*infected	0 (0)
*W. bancrofti*	10	Sri Lanka and Tahiti	*W. bancrofti-*infected	0 (0)
Healthy controls	84	Belgium	No helminth infection	nd
South America	96	Brazil/South-America	No helminth infection (90 *T. cruzi*-seropositive)	nd
STH/*S. mansoni*	24	Ethiopia, Jimma	STH- and/or *S. mansoni-*infected	0 (0)
Endemic controls	50	Ghana, Ashanti	No clinical signs of onchocerciasis	25 (50.0)
Kenya, Kisumu	100	Kenya, Kisumu	45 *S. mansoni-*infected, 3 STH-infected	0 (0)
Kenya, Siaya	108	Kenya, Siaya	43 STH-infected, 2 *S. mansoni*-infected	1 (0.9)

Plasma samples from a region in Kenya were collected as part of a field study in Kenya. This study was undertaken in the former Nyanza Province, in the southwest part of Kenya, with collections in the Kisumu county (high *S. mansoni* prevalence area) and Siaya county (high STH prevalence area). Stool samples were collected in order to determine the STH and *S. mansoni* infection status, based on microscopic egg counting by the Kato-Katz technique. A total of 208 subjects that had donated plasma samples were included in this study.

A set of 90 plasma samples from patients with chronic Chagas cardiomyopathy that were also *Trypanosoma cruzi-*seropositive were provided by InfYnity Biomarkers. A total of 48 samples were obtained from Barcelona’s blood bank from South-American immigrants and 42 samples were collected in Minas Gerais state in Brazil. Additionally, 6 samples from *T. cruzi-*uninfected South American immigrants were obtained from Barcelona’s blood bank.

### Onchocerciasis IgG4 rapid test

The presence of IgG4 antibodies against the *O. volvulus* antigen Ov-16 was determined using the SD BIOLINE Onchocerciasis IgG4 test (Standard Diagnostics, Gyeonggi-do, Republic of Korea), according to the manufacturer’s instructions. Briefly, 10 µl of plasma was added to the round sample well on the lateral flow strip, immediately followed by the addition of 4 drops of assay diluent into the square assay diluent well. After 1 hour, tests were scored. Tests were considered positive only when both the test and control line were visible; faint lines were considered positive, as recommended by the manufacturer.

### Multiplex peptide ELISA

Multiplex peptide ELISA plates were produced by InfYnity Biomarkers, Lyon, France (www.infynity-biomarkers.com). The sciFLEXARRAYER system (Scienion, Dortmund, Germany) was used to print C-terminally biotinylated peptides, linked to streptavidin beforehand, in duplicates in each well of a 96-well plate. In order to identify false positivity due to anti-streptavidin antibodies, a streptavidin control was printed in duplicate. In addition to these antigens, three spots of positive controls (PC) designed to check for the presence of human samples and enzyme conjugates were printed on the array. Additionally, low and medium control spots were embedded. A net intensity (mean value of duplicated spots intensity) score was obtained for each of the measured peptide-specific antibodies. To assess the reproducibility of microplates production, a non-destructive QC was performed consisting of visual inspection of printing consistency throughout the full production cycles.

The microplates were processed following the manufacturer’s instructions using the reagents provided in the kits. Briefly, the plasma samples diluted at 1:100 were incubated for 1 h at room temperature, then washed three times with PBST. The dilution parameter was optimized during the assay development. Next, horseradish peroxidase (HRP)-conjugated goat anti-human IgG antibodies (CliniSciences, Nanterre, France) adequately diluted was added to the microplate for 1 h at room temperature. The microplates were then washed three times before adding a precipitating TMB formulation for 20 min at room temperature in the dark. Then, TMB solution was removed and plates were dried at 37 °C for 10 min. Each plate was then imaged using the sciREADER CL2 (Scienion). The software calculated the pixel intensity for each spot. In order to establish the net intensity for each antigen, we considered the mean value of the paired spots.

### Malaria antibody ELISA

The presence of antibodies against recombinant antigens of *P. falciparum* and *P. vivax* was determined using the anti-malaria kit from Abcam PLC (Cambridge, UK). The tests were performed according to the manufacturer’s instructions using the reagents provided in the kit.

### Statistical analysis

All analyses and plots were generated using the R environment (v3.5.2, https://www.R-project.org/) and R packages *pROC* (v1.13.0) and *ggplot2* (v3.1.0) or GraphPad Prism v8.0.0).

## Results

### The multiplex ELISA

The three peptides with the best performance determined in our previous studies (OvMP-1, OvMP-23 and OvNMP-48) were spotted in the multiplex ELISA system. Reproducibility of the multiplex ELISA was measured using three independent production lots and tested with a total of 480 plasma samples. In total, 3 × 480 data points were used for each peptide to perform multiple linear regression and *r*^2^ values were calculated (Table 2). To avoid false positive results due to streptavidin-binding antibodies, we introduced a technical cut-off of 7.7855 spot intensity by which 95% of samples were found below this value for the streptavidin control spot. A total of 22 samples with signal intensity on the streptavidin control spot ≥ 7.7855 were excluded for further analysis. We concluded that the multiplex peptide ELISA can be produced and performed in a reproducible manner.

**Table 2:** Parameters for *Onchocerca volvulus* peptides in the multiplex ELISA after testing on 480 plasma samples on 3 different production lots

Peptide ID	Sequence	*r*^2^	Intensity (Q1-Median-Q3)	Spot intensity cut-off
OvMP-1	VSV**EPVTTQET**VSV	0.97	4.6–85.4–101.0	17.43
OvMP-23	VSV**KDGEDK**VSV**QTSNLD**VSV	0.98	1.5–23.7–74.5	12.44
OvMP-48	VSV**DNNGANFE**VSV**NLNANSNPN**VSV**EKDGKK**VSV	0.95	0.8–11.7–34.8	8.04

*Note*: Sequences in bold represent the different epitopes. Correlation coefficients *r*^2^ are based on multiple linear regression. The signal intensity levels of the individual peptides represent 1st quartile – median – 3rd quartile. “Spot intensity cut-off” threshold of all individual peptides was calculated based on receiver operating characteristic (ROC) analysis

**Fig. 1:**
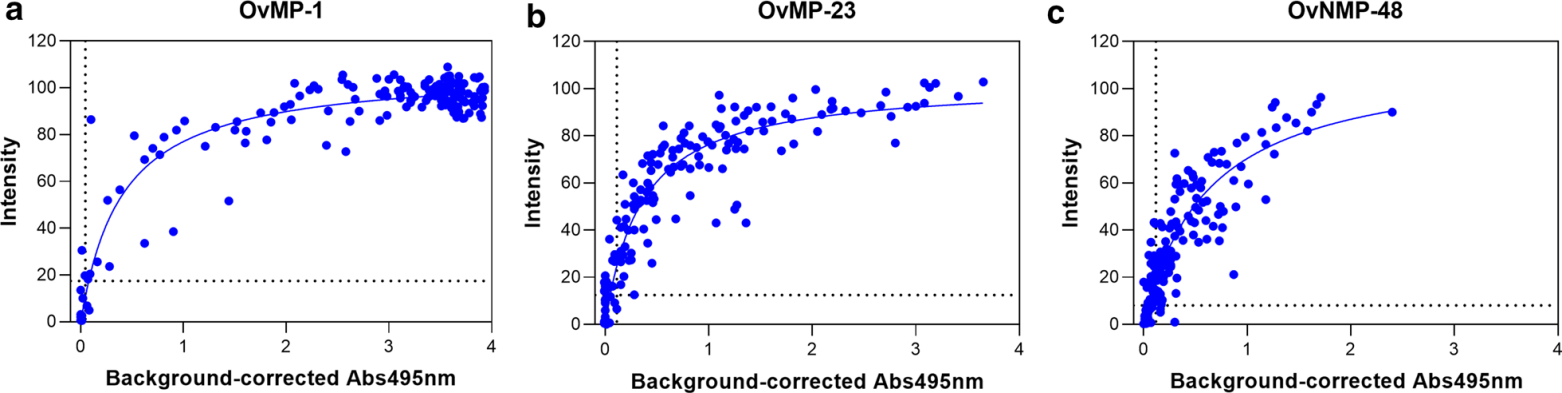
Correlation between spot intensity as measured in the multiplex ELISA and absorbance previously measured in singleplex ELISA for OvMP-1 (**a**), OvMP-23 (**b**) and OvNMP-48 (**c**). Regression lines were calculated using least squares fit. Spot intensity cut-offs are indicated by the horizontal dashed lines; absorbance cut-offs are indicated by the vertical dashed lines [12, 13]

Comparing the results of the multiplex ELISA with the previously published singleplex ELISA [12, 13, 17] showed a correlation between the spot intensities (multiplex assay) and absorbance (singleplex assay). The intensity measured in the multiplex assay rapidly increased with increasing antibody levels and started to saturate at a level that corresponds with an absorbance of 1 (Fig. 1).

**Fig. 2:**
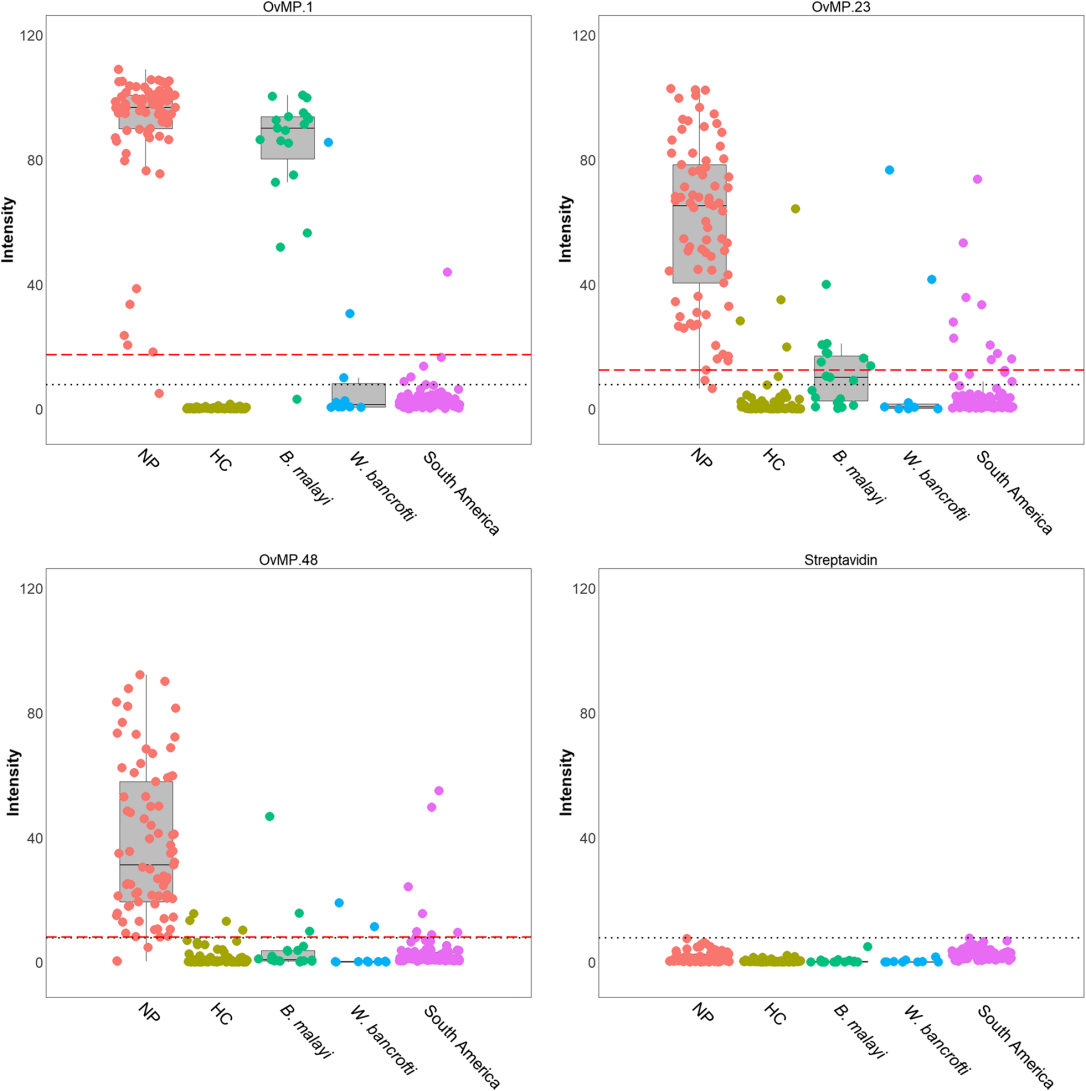
Individual data points and box plots of the samples from individuals with known onchocerciasis status for OvMP-1, OvMP-23, OvNMP-48 and streptavidin spot. Red dotted line indicates the threshold as calculated by ROC analysis, black dotted line is the technical cut-off. Boxplots represent the main statistical characteristics for each sample set. The lower and upper hinges correspond to the first and third quartiles (the 25th and 75th percentiles), the middle line corresponds to the median

Besides the technical streptavidin-based cut-off, for each of the peptides a threshold for positivity was determined using receiver operating characteristic (ROC) analysis with data derived from testing confirmed onchocerciasis patients *versus* a control population from *O. volvulus* non-endemic regions (Fig. 2 and Table 3: known onchocerciasis status). The “spot intensity cut-offs” are given in Table 2. Occasionally, single peptide reactivity above the threshold was seen in control samples. A simple algorithm was designed: samples reactive on 0, 1 or 2 peptides were considered negative for *O. volvulus* infection, while 3-peptide positive reaction was necessary to confirm seropositivity. We concluded that the use of a 3-peptide multiplex ELISA resulted in a sensitivity of 93.5% and a specificity of 99.0%.

**Table 3:** Number of samples (and percentages) found to be positive for the three different peptide serology markers

Sample set	Number (%) of plasma samples above “spot intensity cut-off” for peptide			
	OvMP-1	OvMP-23	OvNMP-48	3-peptide positives
Known onchocerciasis status				
Nodule positive (Ghana, *n* = 77)	76 (98.7)	75 (97.4)	74 (96.1)	72 (93.5)
* B. malayi* (Indonesia, *n* = 19)	18 (94.7)	8 (42.1)	3 (15.8)	1 (5.3)
* W. bancrofti* (Sri Lanka, *n* = 8; Tahiti, *n* = 2)	2 (20.0)	2 (20.0)	2 (20.0)	1 (10.0)
Healthy controls (Belgium, *n* = 84)	0 (0)	4 (4.8)	4 (4.8)	0 (0)
South America (n=89)	1 (1.1)	10 (11.2)	7 (7.9)	0 (0)
Unknown onchocerciasis status				
Kenya, Kisumu (*n* = 98)	86 (87.8)	55 (56.1)	45 (45.9)	30 (30.6)
Kenya, Siaya (*n* = 98)	83 (84.7)	66 (67.3)	69 (70.4)	57 (58.2)
STH/*S. mansoni* (Ethiopia, *n* = 24)	3 (12.5)	5 (20.8)	2 (8.3)	0 (0)
Endemic controls (Ghana, *n* = 48)	46 (95.8)	46 (95.8)	43 (89.6)	40 (83.3)

### Performance of the multiplex peptide ELISA in populations with unknown infection

The seroreactivity against the three peptides was investigated in individuals with unknown *O. volvulus* infection status (Table 3: unknown onchocerciasis status). In agreement with what was published before for singleplex ELISA, most samples from Ethiopia positive for soil-transmitted helminths (STH) and/or *S. mansoni* infection were negative for the peptides, and none were found to be positive for all three peptide markers [18].

In two sample sets collected in an onchocerciasis-free area of Kenya (Kisumu county with high prevalence of *S. mansoni*; and Siaya county with high STH prevalence), more than 80% were positive for OvMP-1, both in Kisumu and Siaya county, and unexpected high seropositivity for OvMP-23 and OvNMP-48 was measured. The 3-peptide positives algorithm would label > 40% seropositive for *O. volvulus* in Kenya, a result that is in strong disagreement with the epidemiology of onchocerciasis. It was concluded that the three peptides bearing linear *O. volvulus* epitopes are recognized by antibodies induced by either/or *O.* *volvulus* and yet another immunogen, but not STH, *S. mansoni*, *W. bancrofti*, *B. malayi*, HIV, HCV, dengue, and possibly also not by the malaria parasite [12, 13, 18]. The majority of the endemic control samples from Ghana were found to be positive for peptides OvMP-1, OvMP-23 and/or OvNMP-48, with 83.3% of them positive for all three peptides.

The presence of antibodies against recombinant antigens of *P. falciparum* and *P. vivax* was determined in the 196 samples from Kenya (Table 4). Since in the 20 samples found to be negative for malaria, 16, 6 and 4 samples were found to be positive for OvMP-1, OvMP-23 and OvNMP-48, respectively, there is no evident causal relationship between infection with *Plasmodium* spp. and an immune response against these peptides.

**Table 4:** Number of samples found to be positive for the three different peptide serology markers in the Kenyan population, stratified according to malaria serological status

Malaria serostatus	n	Number (%) of plasma samples above “spot intensity cut-off” for peptide			
		OvMP-1	OvMP-23	OvNMP-48	3-peptide positives
Negative	20	16 (80.0)	6 (30.0)	4 (20.0)	2 (10.0)
Intermediate	11	11 (100)	7 (63.6)	4 (36.4)	3 (27.3)
Positive	165	142 (86.1)	108 (65.5)	106 (64.2)	82 (49.7)
Total	196	169 (86.2)	121 (61.7)	114 (58.2)	87 (44.4)

## Discussion

In this study, the performance of an InfYnity Biomarkers multiplex peptide ELISA was evaluated for the detection of antibodies against different linear epitopes of *O. volvulus* using a similar approach to what was already applied on Chagas disease serology [15, 16]. The panel of three peptides that was used is based on six different linear epitopes identified previously [12–14, 17, 18]. As previously observed, no IgG4 antibodies against these epitopes were detected, and therefore it was decided to determine epitope-specific total IgG in the multiplex ELISA [14].

First, the comparability of the multiplex and singleplex data was evaluated. Multiplex and singleplex data were comparable for all three peptides. Three independent batches of plates were produced on different days, using different solutions of peptides, and using a different piezo crystal (which pulses an electric current flow, resulting in a drop of the solution out of the nozzle [19]). A set of 480 plasma samples was simultaneously analyzed on the three different batches. For all three peptides a good correlation (*r* ^2^≥ 0.95) was found.

The multiplex ELISA was used to determine seroreactivity against the different *O. volvulus* epitopes in Kenyan individuals with unknown *O. volvulus* infection status. Kenya was the first country that used synthetic insecticides to eliminate the vector that transmits *O.* *volvulus*, in the case of Kenya being *Simulium neavei* [20, 21]. Already in 1955, vector control was completed and follow-up evaluations at 18 years after interruption of transmission demonstrated that microfilariae were no longer found in the skin, and onchocerciasis was considered to be eliminated in Kenya [22–24]. Two sets of plasma samples were collected: one set from Kisumu county (high prevalence of *S. mansoni*), the other set from Siaya county (high prevalence of STH). Only one out of 196 individuals had a positive Ov16 IgG4 response. We concluded that these populations were indeed onchocerciasis-free. In contrast however, 87 out of 196 (44.4%) samples were found to be triple-peptide positive.

Analysis of the evidence for candidates for this unexpected cross-reactivity falls into two groups: organisms to be excluded, and hypothetical candidates. First, the organisms that can be excluded are as follows: (i) STH and *S. mansoni:* individuals with infection from Ethiopia do not respond to the different peptides [18], and this is confirmed in this multiplex ELISA (Table 3); (ii) *W. bancrofti* (causing lymphatic filariasis): is confined to the Kenya coastal region and it is therefore unlikely to cause such high seroprevalence in Kisumu and Siaya counties [25]; (iii) *Plasmodium*: 84.2% of the Kenyan samples were seropositive for *Plasmodium*, while only half of them were triple-peptide positive. Furthermore, most of the 20 samples that were seronegative for *Plasmodium* were seropositive for at least one peptide (2 samples for all 3 peptides), making it unlikely that *Plasmodium* is causing these responses; and (iv) HIV, HCV and dengue virus: previous analyses using singleplex ELISA demonstrated that no response could be detected against any of the three peptides in individuals infected with one of these viruses.

Secondly, hypothetical causes for the cross-reactivity are as follows: (i) exposure to zoonotic *Onchocerca* species: *O.* *gutturosa* or *O. fasciata* can induce a cross-reactive immune response upon exposure [26–28], and cross-reactive antibodies were found in individuals living in areas where *O. ochengi* infects cattle [29]. This parasite requires *Simulium* species which are no longer present in Kenya [20, 21], hence this zoonotic hypothesis is unlikely; (ii) *Mansonella perstans:* probably the most prevalent known human filarial infection [30, 31] and possibly the most neglected filariasis [32]. It is estimated that more than 100 million people may be infected by *M. perstans*, predominantly in sub-Saharan Africa as well as in the northern part of the Amazon rainforest stretching from equatorial Brazil to the Caribbean coast of South America [32]. Historical reports however also indicated the presence of *Mansonella* in the Pacific region [33]. It was indicated before that mansonellosis parasites might interfere with some onchocerciasis immunodiagnostic assays [34]. In countries such as Ghana, Uganda and Cameroon, it was shown that in some communities, prevalence reaches levels above 70% [35–37]. The samples we tested from South America were from a non-endemic region in Brazil (Minas Gerais State) and the fact that most of them were seronegative for the three peptides at least does not contradict the hypothesis that *M.* *perstans* is causing the peptide specific responses. There is no information on mansonellosis in the Kisumu and Siaya counties, but it is thought to be endemic in Kenya [30]; (iii) *Strongyloides stercoralis*: current data indicate that *S. stercoralis* infections affect between 10% and 40% of the population in many tropical and subtropical countries. Community-based surveys in Cameroon, Ghana and Kenya showed a prevalence of 10.0%, 69.5% and 80.2% respectively [38]; and (iv) unknown: certain viral or bacterial infections or environmental stimuli might also induce the immune response directed against the peptides. In this respect, one might consider mining all the proteomes available in the databases. However, as we already demonstrated for OvMP1, OvMP-2 and OvMP-3, there are multiple proteins from several organisms that bear these epitope sequences. Although some of these proteins might even be antigenic in case the epitope is accessible in the native protein, no conclusion can be drawn on the immunogenicity of these protein sequences *in vivo* [14]. Furthermore, peptides may mimic essential features of epitopes (mimotopes) [39]. Therefore, the true immunogenic stimulus that resulted in the antibodies detected here, might even not contain the epitope sequence. Based on these considerations, it is unlikely that this approach will lead to the identification of the true immunogen.

Further evaluation of *O. volvulus* endemic control samples, i.e. from individuals living in a Ghana *O. volvulus* endemic area, but who had no visible signs of onchocerciasis, showed the majority (83%) to be triple-peptide positive, while 50% of these endemic controls were found to be positive for Ov-16 IgG4 antibodies (Table 1). Considering the above observations, we cannot conclude on the exposure status of the endemic control group beyond the Ov16 IgG4 antibody response. The data as currently available do not exclude the possibility that OvMP-1, OvMP–23, and OvNMP-48 react both with antibodies raised against *O. volvulus*, and/or solely to the as-yet to be identified immunogen.

It must be noted that two of the peptides evaluated in this study are designed to cover two or three different epitopes. Since a positive response against such peptide does not discriminate the response against the individual epitopes, there is still the possibility that some of the individual epitopes are specific for *O. volvulus*.

The observed cross-reactivity on the *O. volvulus* derived peptides opens an additional discussion on the quality check of the biobanks used for biomarker discovery activities. Since discovery of these peptides was performed on samples from Cameroon [14] and confirmation of their reactivity was done on samples from Ghana, Indonesia, Sri Lanka [12, 13] and Kenya, we came to the conclusion that, despite the careful selection of samples, we knew little or nothing of other filaria past or present co-infections. Therefore, validation of candidate biomarkers should be performed on extremely well-characterized samples for multiple infections. This would require a holistic view on biobanking, which would include biomaterial collection beyond the study objective. The presented work should also be seen as a call to the NTD community to collaborate precompetitively on biobanking initiatives prior to embarking on the biomarker discovery and validation.

## Conclusions

This study demonstrates that a multiplex ELISA based on three *O. volvulus* peptides was successfully implemented. Assessment of the utility of this serology assay indicates that other, yet to be identified stimuli, significantly cross-react with these peptides. This observation invalidates the usefulness of the peptide serology markers for onchocerciasis detection.

## Data Availability

Data supporting the conclusions of this article are included within the article. The datasets used and/or analyzed during the present study are available from the corresponding author on reasonable request.

## Abbreviations

WHO: World Health Organization
NTD: neglected tropical diseases
MDA: mass drug administration
RDT: rapid diagnostic test
mf: microfilariae
ELISA: enzyme-linked immunosorbent assay
STH: soil-transmitted helminths
